# Correction: Cost-effectiveness evaluations of the 9-Valent human papillomavirus (HPV) vaccine: Evidence from a systematic review

**DOI:** 10.1371/journal.pone.0294379

**Published:** 2023-11-09

**Authors:** Rashidul Alam Mahumud, Khorshed Alam, Syed Afroz, Gail M. Ormsby, Jeff Dunn, Jeff Gow

There is an error in the affiliations for the first author. Specifically, #3 was included in error and should not have been indicated. The correct affiliations are as follows:

Rashidul Alam Mahumud^1,2,3,4*^, Khorshed Alam^1,2^, Syed Afroz Keramat^1,2,5^, Gail M. Ormsby^6^, Jeff Dunn^1,7,8^, Jeff Gow^1,2,9^

**1** Health Economics and Policy Research, Centre for Health Research, University of Southern Queensland, Toowoomba, Queensland, Australia, **2** School of Commerce, University of Southern Queensland, Toowoomba, Queensland, Australia, **3** School of Social Sciences, Western Sydney University, Penrith, Australia, **4** Translational Health Research Institute, Western Sydney University, Penrith, Australia, **5** Economics Discipline, School of Social Science, University of Khulna, Khulna, Bangladesh, **6** School of Health and Wellbeing, University of Southern Queensland, Toowoomba, Queensland, Australia, **7** Cancer Research Centre, Cancer Council Queensland, Fortitude Valley, Queensland, Australia, **8** Prostate Cancer Foundation of Australia, St Leonards, NSW, Australia, **9** School of Accounting, Economics and Finance, University of KwaZulu-Natal, Durban, South Africa

Additionally, there was an error in the corresponding author’s listed email addresses. Correspondence regarding this article should only be sent to the following email address: rashed.mahumud@usq.edu.au.
