# Supplementary material for: Cost-effectiveness evaluations of the 9-Valent human papillomavirus (HPV) vaccine: Evidence from a systematic review
Source: PLoS One. 2020 Jun 2;15(6):e0233499. doi: 10.1371/journal.pone.0233499 (PMC7266321; doi:10.1371/journal.pone.0233499)
Supplement: S1 Checklist — (DOC) [file pone.0233499.s001.doc]

| Section/topic | # | Checklist item | Reported on page # |
| --- | --- | --- | --- |
| TITLE | | |  |
| Title | 1 | *Cost-effectiveness evaluations of the 9-Valent human papillomavirus (HPV) vaccine: Evidence from a systematic review* | 1 |
| ABSTRACT | | |  |
| Structured summary | 2 | **Introduction**  The World Health Organization (WHO) recommends that programs for human papillomavirus (HPV) vaccination are established to be cost-effective before implementation. HPV vaccination is WHO recommended for girls aged 9–13 years old due to the high burden of cervical cancer. This review examined evidence of the cost-effectiveness evaluation of the 9-valent HPV vaccine within a global context.  **Methods**  Searches were performed until 31 July 2019 using two databases: PubMed and Scopus. A combined checklist (i.e., WHO, Drummond and CHEERS) was used to examine the quality of eligible studies. A total of 12 studies were eligible for review and nearly all were conducted in developed countries.  **Results**  Despite some heterogeneity in approaches to measuring cost-effectiveness, ten studies concluded that 9vHPV vaccination was cost-effective while two studies were not. The addition of adolescent boys into immunisation program was cost effective when vaccine price and coverage was comparatively low. When vaccination coverage for female was more than 75%, gender neutral HPV vaccination was less cost-effective than when targeting only girls aged 9–18 years. Multi cohort immunization approach was cost-effective in the age range of 9–14 years but the upper age limit at which vaccination was no longer cost-effective requires to be further evaluated. Most dominating parameters determined were duration of vaccine protection, time horizon, vaccine price, coverage, healthcare costs, efficacy and discounting rates.  **Conclusions**  These findings are anticipated to support policy-makers in extending HPV immunization programs on either switching to the 9-valent vaccine or the inclusion of adolescent boys’ vaccination or extending the age range for vaccination. Further, this review also supports extending vaccination to low-resource settings where vaccine prices are competitive, donor funding is available, the cervical cancer burden is high or screening options are limited. | 1 |
| INTRODUCTION | | |  |
| Rationale | 3 | Cervical cancer (CC) is both a leading cancer and the leading cause of cancer deaths in women globally [1]. Approximately 570,000 new cases of CC were diagnosed in 2018, composing 6.6% of all cancers in women [1]. The burden of CC is an alarming issue worldwide, especially in low- and middle-income countries (LMICs). Approximately 85% of CC cases and 90% of deaths from CC occur in LMICs [1]. Persistent infections with human papillomavirus (HPV) are a key cause of CC and is an established carcinogen of CC [2]. HPV is predominantly transmitted to reproductive-aged women through sexual contact [3]. Most HPV infections are transient and can be cured within a short duration, usually a few months after their acquisition. However, untreated HPV infections can continue and evolve into cancer in some cases. There are more than 100 types of HPV infections, and high-risk types develop into CC [4]. Thirteen high-risk HPV genotypes are known to be predominantly responsible for malignant and [premalignant](https://www.synonyms.com/synonym/premalignant) lesions of the anogenital area [5], and these are the leading causes of most aggressive CC [6]. Further, HPV is also responsible for the majority of anogenital cervical cancers, including anal cancers (88%), vulvar cancers (43%), invasive vaginal carcinomas (70%), and all penile cancers (50%) globally [4].  The burden of CC (i.e., high incidence and mortality rates) globally is preventable through the implementation of a primary prevention strategy such as vaccination [1]. There are vaccines that can protect common cancer-causing types of HPV and reduce the risk of CC significantly. Three types of HPV vaccines, namely bivalent (Cervarix), quadrivalent (Gardasil) and 9-valent vaccine (Gardasil-9), are currently available in the market. Unfortunately, as of March 2017, only 71 countries (37% of all countries) have introduced HPV vaccines in their national immunization programs for girls, and 11 countries (6%) for both sexes [2]. The first global recommendation on HPV vaccination was proposed by the World Health Organization’s Strategic Advisory Group of Experts on Immunization in October 2008 [7], whereby HPV vaccination was recommended for girls aged 9–13 years old. This recommendation was updated in April 2014 [8], with the emphasis to include extended 2-dose HPV immunization for girls aged 9–14 years, who were not immunocompromised. With the recent licensing of the 9-valent vaccine and the introduction of various HPV vaccination strategies, an update on the current recommendations of HPV vaccination are inevitable. The goals of the immunisation program are to reduce the acquisition and spread of HPV infections and to achieve optimum coverage through effective delivery systems. According to the underlying distribution of HPV infection types of CC, the 9vHPV vaccine builds population-level strong immunity against HPV-6, 11, 16, 18, 31, 33, 45, 52, and 58 infections [5] that cumulatively contribute approximately 89% of all CCs globally [9]. Considering the primary prevention of HPV infection, the 9vHPV vaccine is expected to reduce by an additional 10% the lifetime risk of diagnosis with CC in immunised cohorts compared with the 4vHPV vaccine and reduce CC by an additional 52% in non-vaccinated cohorts [10]. | 2-3 |
| Objectives | 4 | This review aims to update current evidence on the economic viability of HPV vaccination. In addition, this study aims to examine the cost-effectiveness of the 9-valent vaccine when boys are included and when age cohorts are varied, all within a global context. This review may be used as comprehensive evidence of general trends on the ongoing cost-effectiveness evaluation of HPV vaccine. | 3 |
| METHODS | | |  |
| Protocol and registration | 5 | The protocol has been submitted to PROSPERO, Centre for Reviews and Dissemination, University of York. [https://www.crd.york.ac.uk/PROSPERO/#recordDetails](https://www.crd.york.ac.uk/PROSPERO/#recordDetails). This protocol is under review that it is being assessed by the editorial team. | Under Review |
| Eligibility criteria | 6 | Three authors of the review team independently examined the titles and abstracts of the articles that met the selection criteria. The existing academic literature in the cost-effectiveness of 9-valent HPV vaccination was searched. Exclusion of articles was based on: ‘not cost-effectiveness analyses, ‘insufficient cost and cost-effectiveness related data’, or ‘not using nine-valent HPV vaccine’. Language restrictions were not applied. | 4 |
| Information sources | 7 | The literature search was performed by searching Scopus and PubMed to identify relevant articles following the inclusion criteria. | 4 |
| Search | 8 | Search inclusion terms included ‘economic evaluation’, ‘cost-effectiveness’, ‘analysis’, ‘human papillomavirus’, ‘HPV’, ‘vaccine’, ‘vaccinated’, ‘vaccination’, ‘cervical cancer’, ‘non-valent’, ‘9 or nine-valent’. We have started searching of articles on 27^th^ September 2019 and ending on 31^st^ July 2019. | 4 |
| Study selection | 9 | Three authors of the review team independently examined the titles and abstracts of the articles that met the selection criteria (e.g., screening, eligibility), included in systematic review. The existing academic literature in the cost-effectiveness of 9-valent HPV vaccination was searched. Exclusion of articles was based on: ‘not cost-effectiveness analyses, ‘insufficient cost and cost-effectiveness related data’, or ‘not using nine-valent HPV vaccine’. Language restrictions were not applied. Four hundred and eighty one articles were yielded through the primary search, of which 78 articles were discarded because of duplication. Fifty one articles were considered for full-text review after screening by title and abstract. Of these, 12 articles were eligible for the final review. Three hundred fifty-two articles were excluded from this study following the inclusion criteria. The reasons for exclusion were: conference abstract (n = 58), reviews or editorials or commentary (n = 160), not cost-effectiveness evaluations (n = 60), did not use 9-valent vaccine (4v-HPV, 2v-HPV; n = 72) and insufficient information (n = 2). Finally, 12 articles were included in this review. | 4-5 |
| Data collection process | 10 | The study strategy followed a number of checks to ensure consistency of approach, including a discussion about discrepancies within the study team. For each outcome and model input parameters, the authors identified the proportion of missing observations and compare them with data in the original publication. In addition, a range of checks was carried out for all included studies to ensure that all values were reasonable. Datasets were combined to form a new master dataset where model input assumptions and outcome-related parameters used in the original studies were included. Further, two authors independently assessed the analytical quality of the preliminary selected studies using appropriate tools for examining risk of bias. Disagreements on inclusions were resolved by discussion with a third review author. | 4 |
| Data items | 11 | The study selection process was conducted in line with the PRISMA guidelines [11]. Data extraction was performed to develop a comprehensive data matrix which summarises the study characteristics such as authors, settings, perspective, threshold, outcome-related parameters and other necessary information. Two authors (RAM and SAK) independently reviewed the titles and abstract. Data from all eligible studies were extracted by the same two authors using a standardized data collection form. A matrix was developed to summarise the characteristics and findings of the studies. Studies were characterized by incorporating four themes: (i) study used 9-valent HPV vaccine to examine the cost-effectiveness, (ii) target population demographic characteristics (e.g., gender-neutral and multiple age cohort immunisation), (iii) study perspectives, model and economic level of each country, and (iv) model input and outcome-related parameters. The review showed evidence in terms of methodological and current practices of cost-effectiveness evaluation studies such as determination of study research questions; the study perspective adopted, the duration of vaccine protection, time horizon and discount rate; explanation of model performed for data analysis; model input assumptions behind the estimation of associated costs and outcome parameters; reporting of ICERs; most dominant parameters of sensitivity analysis; examination of study conclusions and recommendations as well as financial disclosure of the selected studies. | 4-6 |
| Risk of bias in individual studies | 12 | Risk of bias was examined using the Consensus Health Economic Criteria (CHEC) list, a checklist that can be used to critically evaluate published economic evaluations [12].The study strategy followed a number of checks to ensure consistency of approach, including a discussion about discrepancies within the study team. For each outcome and model input parameters, the authors identified the proportion of missing observations and compare them with data in the original publication. In addition, a range of checks was carried out for all included studies to ensure that all values were reasonable. Datasets were combined to form a new master dataset where model input assumptions and outcome-related parameters used in the original studies were included. Further, two authors independently assessed the analytical quality of the preliminary selected studies using appropriate tools for examining risk of bias. Disagreements on inclusions were resolved by discussion with a third review author. | 4 |
| Summary measures | 13 | Incremental cost-effectiveness ratio (ICERs), DALY averted, QALY gained, case averted, death averted | 5 |
| Synthesis of results | 14 | Two authors (RAM and SAK) independently reviewed the titles and abstract. Data from all eligible studies were extracted by the same two authors using a standardized data collection form. A matrix was developed to summarise the characteristics and findings of the studies. Studies were characterized by incorporating four themes: (i) study used 9-valent HPV vaccine to examine the cost-effectiveness, (ii) target population demographic characteristics (e.g., gender-neutral and multiple age cohort immunisation), (iii) study perspectives, model and economic level of each country, and (iv) model input and outcome-related parameters.  To compare findings across the selected studies, incremental cost-effectiveness ratios (ICERs) and standardized cost-effectiveness were outlined. In terms of standardized cost-effectiveness scenarios, these studies used the heuristic cost-effectiveness threshold guided by the WHO [13], wherein an intervention or program was evaluated to be cost-effective if the ICER/DALYs averted was less than three times a country’s annual per capita Gross Domestic Product (GDP). Further, the WHO constructed three broad decision rules: (i) an intervention or program was recommended as very cost-effective if ICER/DALYs averted <1 time GDP threshold; (ii) cost-effective if ICER/DALYs averted ≥ 1 time GDP threshold and ≤ 3 times GDP threshold; and (iii) not cost-effective if ICER/DALYs averted >3 times GDP threshold [14]. Examining whether an ICER offered by any strategy signifies value for money requires comparison to a cost-effectiveness threshold (CET). The CET refers to the health effects foregone (i.e., opportunity costs) related to resources being devoted to an intervention and consequentially being unavailable for other health-care priorities. Policy makers should be willing to invest their limited resources in the strategy offering the greatest health gains. CETs for the country with the lowest income in the world, borderline low/low-middle income, borderline low-middle/upper-middle income, and borderline high-middle/high income were estimated to be 1% to 51% GDP per capita, 4% to 51%, 11% to 51%, and 32% to 59%, respectively [15].  The review showed evidence in terms of methodological and current practices of cost-effectiveness evaluation studies such as determination of study research questions; the study perspective adopted, the duration of vaccine protection, time horizon and discount rate; explanation of model performed for data analysis; model input assumptions behind the estimation of associated costs and outcome parameters; reporting of ICERs; most dominant parameters of sensitivity analysis; examination of study conclusions and recommendations as well as financial disclosure of the selected studies. | 5 |

Page 1 of 2

| **Section/topic** | **#** | **Checklist item** | **Reported on page #** |
| --- | --- | --- | --- |
| Risk of bias across studies | 15 | The study strategy followed a number of checks to ensure consistency of approach, including a discussion about discrepancies within the study team. For each outcome and model input parameters, the authors identified the proportion of missing observations and compare them with data in the original publication. In addition, a range of checks was carried out for all included studies to ensure that all values were reasonable. Datasets were combined to form a new master dataset where model input assumptions and outcome-related parameters used in the original studies were included. Further, three authors independently assessed the analytical quality of the preliminary selected studies using appropriate tools for examining risk of bias. Disagreements on inclusions were resolved by discussion with a third review author. |  |
| Additional analyses | 16 | Not applicable | Not applicable |
| **RESULTS** | | |  |
| Study selection | 17 | Four hundred and eighty one articles were yielded through the primary search, of which 78 articles were discarded because of duplication. Fifty one articles were considered for full-text review after screening by title and abstract. Of these, 12 articles were eligible for the final review. Three hundred fifty-two articles were excluded from this study following the inclusion criteria. The reasons for exclusion were: conference abstract (n = 58), reviews or editorials or commentary (n = 160), not cost-effectiveness evaluations (n = 60), did not use 9-valent vaccine (4v-HPV, 2v-HPV; n = 72) and insufficient information (n = 2). Finally, 12 articles were included in this review. | 6 |
| Study characteristics | 18 | Please see Table 1-3. | Please see Table 1-3 |
| Risk of bias within studies | 19 | Not applicable | Not applicable |
| Results of individual studies | 20 | For all outcomes considered as economically viable of 9-valent vaccine across the countries. | 8-9 |
| Synthesis of results | 21 | Please see Table 3 | Table 3 |
| Risk of bias across studies | 22 | Not applicable |  |
| Additional analysis | 23 | Not applicable |  |
| **DISCUSSION** | | |  |
| Summary of evidence | 24 | The HPV vaccination is one of the cornerstones of CC prevention worldwide. This study explored the cost-effectiveness of 9-valent HPV vaccination, drawing on 12 cost-effectiveness evaluations in order to inform and expand knowledge of the potential influence of the next generation of HPV vaccines. Most studies were conducted in developed countries while one study was performed in an LMIC. However, in the context of LMICs, the incidence of cervical cancer is an alarming public health concern, which warrants an increase in studies which can be extremely useful to influence local decision making [16]. The economic viability of gender-neutral 9-valent HPV vaccination was confirmed by three of the selected studies [17–19]. Cost-effectiveness exploration depends on the coverage of vaccination from the perspective of gender. For example, if the vaccine coverage for female recipients is 80% or above, the majority of the anogenital CC include vulvar cancers, invasive vaginal carcinomas cancers in female could be prevented. As a result, introduction of 9-valent vaccination for boys is relatively less important compared with girls due to the high economic costs involved without the additional benefits gained as per the female population reduction in CC, both from the societal and health system perspectives. Therefore, achieving optimal coverage of vaccination in females should remain a priority. This is of primary significance for LMICs settings since it is more effective and economically viable to prevent CC in females. However, it is also important to note that past studies paid little attention to the broader benefits of vaccination among male cohorts to prevent penile, anal, and oropharyngeal cancers. Exclusion of these diseases related to males may undermine the effectiveness of reducing CC. Gender-neutral vaccination might have several benefits including herd protection for boys. Moreover, it may provide indirect protection to unvaccinated women and direct protection to homosexual men. Therefore, this vaccination strategy should be further considerated in country-level immunization programs by underlining other parameters including disease burden, sexual behaviour in a country (e.g., homosexual intercourse), equity, budget impact, and affordability. | 9-10 |
| Limitations | 25 | This review has some limitations. The cost-effectiveness evaluation based on GDP based thresholds of 1–3 times of GDP per capita lacks country specificity and has little meaning for country-level decision making [20]. It is uncertain whether this threshold truly reflects the country’s affordability or societal willingness to pay for additional health gains. Additionally, GDP is originally intended to measure the experience of people residing in urban areas and thus, it may not actually reflect the experience of the entire population in a country, especially those living in rural areas. Apart from an economic standpoint, other factors should be considered for the national immunization program, such as budget availability, political issues, cultural influences and availability of healthcare workforce. | 12 |
| Conclusions | 26 | Current evidence does not show conclusive proof of greater cost-effectiveness of the new 9-valent vaccine. The inclusion of adolescent males in HPV vaccination programs is cost-effective if vaccine price or coverage of females is low and if the HPV-associated male diseases are also considered. Multiple age cohort vaccination strategy is likely to be cost-effective in the age range of 9–14 years, but the upper age limit at which HPV vaccination is no longer cost-effective needs to be further evaluated. Vaccine coverage, price, duration of protection and discount rates are important parameters for consideration in the uptake of HPV vaccination. Nonetheless, present study findings may be used as an evidence to policy-makers and healthcare providers in making recommendations for HPV national immunization programs on the new 9-valent vaccine or inclusion of adolescent boys’ vaccination or extending the age of immunization, but it should not divert resources from vaccinating the primary target population of girls aged 12 years or from effective cervical cancer screening programs. | 12 |
| **FUNDING** | | |  |
| Funding | 27 | This study was conducted without any financial support. |  |

*From:*  Moher D, Liberati A, Tetzlaff J, Altman DG, The PRISMA Group (2009). Preferred Reporting Items for Systematic Reviews and Meta-Analyses: The PRISMA Statement. PLoS Med 6(7): e1000097. doi:10.1371/journal.pmed1000097

For more information, visit: **www.prisma-statement.org**.

Page 2 of 2

**References**

1. World Health Organization (WHO). National cancer contro programmes: Cervical cancer statistics. 2019. https://www.who.int/cancer/prevention/diagnosis-screening/cervical-cancer/en/. Accessed 29 Aug 2019.

2. Forman D, Lortet-Tieulent J, de Martel C, Ferlay J, Franceschi S, Plummer M, et al. Global burden of human papillomavirus and related diseases. Vaccine. 2012;30:F12–23. doi:10.1016/j.vaccine.2012.07.055.

3. Centers for Disease Control (CDC). Genital HPV infection—fact sheet. Centers for disease control and prevention. 2015. http://www.cdc.gov/std/hpv/stdfact-hpv.htm. Accessed 14 Feb 2019.

4. Mennini FS, Bonanni P, Bianic F, Waure C, Baio G, Plazzotta G, et al. Cost-effectiveness analysis of the nine-valent HPV vaccine in Italy. Cost Eff Resour Alloc. 2017;15:1–14.

5. Guan P, Howell-Jones R, Li N, Bruni L, De Sanjosé S, Franceschi S, et al. Human papillomavirus types in 115,789 HPV-positive women: A meta-analysis from cervical infection to cancer. Int J Cancer. 2012;131:2349–59.

6. Li N, Franceschi S, Howell-Jones R, Snijders PJF, Clifford GM. Human papillomavirus type distribution in 30,848 invasive cervical cancers worldwide: Variation by geographical region, histological type and year of publication. Int J Cancer. 2011;128:927–35.

7. World Health Organization (WHO). Human papillomavirus vaccines: WHO position paper. Wkly Epidemiol Rec. 2009;84:118–31.

8. World Health Organization (WHO). Human papillomavirus vaccines: WHO position paper, October 2014. Wkly Epidemiol Rec. 2014;89:465–91.

9. Serrano B, Alemany L, Tous S, Bruni L, Clifford GM, Weiss T, et al. Potential impact of a nine-valent vaccine in human papillomavirus related cervical disease. Infect Agent Cancer. 2012;7:1–13.

10. Simms KT, Laprise JF, Smith MA, Lew J Bin, Caruana M, Brisson M, et al. Cost-effectiveness of the next generation nonavalent human papillomavirus vaccine in the context of primary human papillomavirus screening in Australia: A comparative modelling analysis. Lancet Public Heal. 2016;1:e66–75. doi:10.1016/S2468-2667(16)30019-6.

11. Moher Bd, Liberati A, Tetzlaff J, Altman DG. Preferred reporting items for systematic reviews and meta-analyses: The PRISMA statement. PLoS Med. 2009;6:e1000097.

12. Evers S, Goossens M, de Vet H, van Tulder M, Ament A. Criteria list for assessment of methodological quality of economic evaluations: Consensus on Health Economic Criteria. Int J Technol Assess Health Care. 2005;21:240–5.

13. WHO Commission on Macroeconomics and Health. Macroeconomics and health: Investing in health for economic development. Report of the Commission on Macroeconomics and Health. 20 Avenue Appia, 1211 Geneva 27, Switzerland; 2001. http://whqlibdoc.who.int/publications/2001/924154550x.pdf.

14. Edejer TT-T, Baltussen R, Adam T, Hutubessy R, Acharya A, Evans D., et al. Making choices in health: WHO guide to cost-effective analysis. 20 Avenue Appia, 1211 Geneva 27, Switzerland; 2003.

15. Woods B, Revill P, Sculpher M, Claxton K. Country-level cost-effectiveness thresholds: Initial estimates and the need for further research. Value Heal. 2016;19:929–35.

16. Thiboonboon K, Santatiwongchai B, Chantarastapornchit V, Rattanavipapong W, Teerawattananon Y. A systematic review of economic evaluation methodologies between resource-limited and resource-rich countries: A case of rotavirus vaccines. Appl Health Econ Health Policy. 2016;14:659–72.

17. Boiron L, Joura E, Largeron N, Prager B, Uhart M. Estimating the cost-effectiveness profile of a universal vaccination programme with a nine-valent HPV vaccine in Austria. BMC Infect Dis. 2016;16:1–15. doi:10.1186/s12879-016-1483-5.

18. Brisson M, Laprise JF, Chesson HW, Drolet M, Malagón T, Boily MC, et al. Health and economic impact of switching from a 4-Valent to a 9-valent HPV vaccination program in the United States. J Natl Cancer Inst. 2016;108:1–9.

19. Chesson HW, Markowitz LE, Hariri S, Ekwueme DU, Saraiya M. The impact and cost-effectiveness of nonavalent HPV vaccination in the United States: Estimates from a simplified transmission model. Hum Vaccines Immunother. 2016;12:1363–72. doi:10.1080/21645515.2016.1140288.

20. Bertram MY, Lauer JA, De Joncheere K, Edejer T, Hutubessy R, Kieny MP, et al. Cost-effectiveness thresholds: Pros and cons. Bull World Health Organ. 2016;94:925–30.
