# Supplementary material for: Cost-effectiveness evaluations of the 9-Valent human papillomavirus (HPV) vaccine: Evidence from a systematic review
Source: PLoS One. 2020 Jun 2;15(6):e0233499. doi: 10.1371/journal.pone.0233499 (PMC7266321; doi:10.1371/journal.pone.0233499)
Supplement: S1 Appendix — (DOCX) [file pone.0233499.s002.docx]

**Appendix A. Search strategies**

**PubMed**

#1

(hpv[Title] OR papilloma⁄[Title] OR cervi⁄[Title]) AND (vaccine⁄ OR vaccinated OR vaccination OR vaccinated OR immune⁄) AND (non-valent/ OR 9 or nine-valent/) AND (cost[Title/Abstract] OR costs[Title/ Abstract] OR cost-effective⁄ OR cost-utility⁄ OR cost-benefit⁄) AND (analysis OR economic evaluation⁄) AND (cervical cancer).

OR

#2 (((cost-effectiveness analysis OR cost-benefit analysis OR cost-utility analysis OR economic evaluation) AND (cervical cancer) AND (vaccine OR vaccination) AND (human papillomavirus OR HPV))) OR ((hpv[Title] OR papilloma⁄ [Title] OR cervi⁄[Title]) AND (vaccine⁄ OR vaccinated OR vaccination OR vaccinated OR immune⁄) AND (non-valent/ OR 9 or nine-valent/) AND (cost[Title/ Abstract] OR costs[Title/Abstract] OR cost-effective⁄ OR cost-utility⁄ OR cost-benefit⁄) AND (analysis OR economic evaluation⁄) AND cervical cancer)

**Scopus**

# 1

TITLE-ABS-KEY ("Economic evaluation" OR "Cost-effectiveness" OR "Cost-benefit analysis" OR "Cost-utility analysis" OR "Analysis" OR "Human papillomavirus" OR "HPV" OR "Vaccine" OR "Vaccinated" OR "Vaccination" OR "Cervical cancer" AND "non-valent" OR "9-Valent"))

# 2

TITLE-ABS-KEY("Economic evaluation" OR "Cost-effectiveness" OR "Cost-benefit analysis" OR "Cost-utility analysis" OR "Analysis" OR "Human papillomavirus" OR "HPV" OR "Vaccine" OR "Vaccinated" OR "Vaccination" OR "Cervical cancer" AND "non-valent" OR "9-Valent" ) AND ( LIMIT-TO ( PUBYEAR,2019) OR LIMIT-TO ( PUBYEAR,2018) OR LIMIT-TO ( PUBYEAR,2017) OR LIMIT-TO ( PUBYEAR,2016) OR LIMIT-TO ( PUBYEAR,2015) OR LIMIT-TO ( PUBYEAR,2014) OR LIMIT-TO ( PUBYEAR,2013) OR LIMIT-TO ( PUBYEAR,2012) OR LIMIT-TO ( PUBYEAR,2011) OR LIMIT-TO ( PUBYEAR,2010) OR LIMIT-TO ( PUBYEAR,2009) OR LIMIT-TO ( PUBYEAR,2008) OR LIMIT-TO ( PUBYEAR,2007) OR LIMIT-TO ( PUBYEAR,2006) OR LIMIT-TO ( PUBYEAR,2005) OR LIMIT-TO ( PUBYEAR,2004) OR LIMIT-TO ( PUBYEAR,2003) OR LIMIT-TO ( PUBYEAR,2002) OR LIMIT-TO ( PUBYEAR,2001) OR LIMIT-TO ( PUBYEAR,2000) )

# 3

TITLE-ABS-KEY ( "Economic evaluation"  OR  "Cost-effectiveness"  OR  "Cost-benefit analysis"  OR  "Cost-utility analysis"  OR  "Analysis"  OR  "Human papillomavirus"  OR  "HPV"  OR  "Vaccine"  OR  "Vaccinated"  OR  "Vaccination"  OR  "Cervical cancer"  AND  "non-valent"  OR  "9-Valent" )  AND  ( LIMIT TO ( PUBYEAR ,  2019 )  OR  LIMIT-TO ( PUBYEAR ,  2018 )  OR  LIMIT-TO ( PUBYEAR ,  2017 )  OR  LIMIT-TO ( PUBYEAR ,  2016 )  OR  LIMIT-TO ( PUBYEAR ,  2015 )  OR  LIMIT-TO ( PUBYEAR ,  2014 )  OR  LIMIT-TO ( PUBYEAR ,  2013 )  OR  LIMIT-TO ( PUBYEAR ,  2012 )  OR  LIMIT-TO ( PUBYEAR ,  2011 )  OR  LIMIT-TO ( PUBYEAR ,  2010 )  OR  LIMIT-TO ( PUBYEAR ,  2009 )  OR  LIMIT-TO ( PUBYEAR ,  2008 )  OR  LIMIT-TO ( PUBYEAR ,  2007 )  OR  LIMIT-TO ( PUBYEAR ,  2006 )  OR  LIMIT-TO ( PUBYEAR ,  2005 )  OR  LIMIT-TO ( PUBYEAR ,  2004 )  OR  LIMIT-TO ( PUBYEAR ,  2003 )  OR  LIMIT-TO ( PUBYEAR ,  2002 )  OR  LIMIT-TO ( PUBYEAR ,  2001 )  OR  LIMIT-TO ( PUBYEAR ,  2000 ) )  AND  ( LIMIT-TO ( DOCTYPE ,  "ar" ) )
