# Supplementary material for: Cost-effectiveness evaluations of the 9-Valent human papillomavirus (HPV) vaccine: Evidence from a systematic review
Source: PLoS One. 2020 Jun 2;15(6):e0233499. doi: 10.1371/journal.pone.0233499 (PMC7266321; doi:10.1371/journal.pone.0233499)
Supplement: Attachment — Submitted filename: PONE-D-19-28575_reviewer A.T.L.pdf [file pone.0233499.s004.pdf]

PLOS ONE

Mapping of cost-effectiveness evaluations of the 9-Valent human papillomavirus (HPV) vaccine: Evidence from a systematic review

--Manuscript Draft--

**Manuscript Number:**	PONE-D-19-28575
**Article Type:**	Research Article
**Full Title:**	Mapping of cost-effectiveness evaluations of the 9-Valent human papillomavirus (HPV) vaccine: Evidence from a systematic review
**Short Title:**	Mapping of cost-effectiveness evaluations of the 9-Valent human papillomavirus (HPV) vaccine
**Corresponding Author:**	Rashidul Alam Mahumud, MPH, MSc, PhD Fellow (Health Economics)<br>International Centre for Diarrhoeal Disease Research<br>Dhaka, Dhaka District BANGLADESH
**Keywords:**	Human papillomavirus; Cost-effectiveness evaluations; Incremental cost-effectiveness ratios; 9-Valent HPV vaccine; systematic review; cervical cancer; World Health Organization
**Abstract:**	Introduction<br>The World Health Organization (WHO) recommends that programs for human papillomavirus (HPV) vaccination are established to be cost-effective before implementation. HPV vaccination is WHO recommended for girls aged 9–13 years old due to the high burden of cervical cancer. This review examined evidence of the cost-effectiveness evaluation of the 9-valent HPV vaccine within a global context.<br>Methods<br>Searches were performed until 31 July 2019 using two databases: PubMed and Scopus. A combined checklist (i.e., WHO, Drummond and CHEERS) was used to examine the quality of eligible studies. A total of 12 studies were eligible for review and nearly all were conducted in developed countries.<br>Results<br>Despite some heterogeneity in approaches to measuring cost-effectiveness, ten studies concluded that 9vHPV vaccination was cost-effective while two studies were not. The addition of adolescent boys into immunisation program was cost effective when vaccine price and coverage was comparatively low. When vaccination coverage for female was more than 75%, gender neutral HPV vaccination was less cost-effective than when targeting only girls aged 9–18 years. Multi cohort immunization approach was cost-effective in the age range of 9–14 years but the upper age limit at which vaccination was no longer cost-effective requires to be further evaluated. Most dominating parameters determined were duration of vaccine protection, time horizon, vaccine price, coverage, healthcare costs, efficacy and discounting rates.<br>Conclusions<br>These findings are anticipated to support policy-makers in extending HPV immunization programs on either switching to the 9-valent vaccine or inclusion of adolescent boys' vaccination or extending the age of vaccination. Further, this review also supports extending vaccination to low-resource settings where vaccine prices are competitive, donor funding is offered, cervical cancer burden is high and screening options are limited.
**Order of Authors:**	Rashidul Alam Mahumud, MPH, MSc, PhD Fellow (Health Economics)
	Khorshed Alam
	Syed Afroz Keramat
	Gail Ormsby
	Jeff Dunn
	Jeff Gow
**Additional Information:**	
**Question**	**Response**

Powered by Editorial Manager® and ProduXion Manager® from Aries Systems Corporation

**Financial Disclosure**<br><br>Enter a financial disclosure statement that describes the sources of funding for the work included in this submission. Review the submission guidelines for detailed requirements. View published research articles from *PLOS ONE* for specific examples.<br><br>This statement is required for submission and **will appear in the published article** if the submission is accepted. Please make sure it is accurate.<br><br>**Unfunded studies**<br>Enter: *The author(s) received no specific funding for this work.*<br><br>**Funded studies**<br>Enter a statement with the following details:<br>• Initials of the authors who received each award<br>• Grant numbers awarded to each author<br>• The full name of each funder<br>• URL of each funder website<br>• Did the sponsors or funders play any role in the study design, data collection and analysis, decision to publish, or preparation of the manuscript?<br>• **NO** - Include this sentence at the end of your statement: *The funders had no role in study design, data collection and analysis, decision to publish, or preparation of the manuscript.*<br>• **YES** - Specify the role(s) played.<br><br>* typeset	The author(s) received no specific funding for this work.
**Competing Interests**<br><br>Use the instructions below to enter a competing interest statement for this submission. On behalf of all authors, disclose any competing interests that could be perceived to bias this work—acknowledging all financial support and any other relevant financial or non-financial competing interests.<br><br>This statement **will appear in the published article** if the submission is accepted. Please make sure it is	The authors have declared that no competing interests exist.

accurate. View published research articles from *PLOS ONE* for specific examples.<br><br>**NO authors have competing interests**<br><br>Enter: *The authors have declared that no competing interests exist.*<br><br>**Authors with competing interests**<br><br>Enter competing interest details beginning with this statement:<br><br>*I have read the journal's policy and the authors of this manuscript have the following competing interests: [insert competing interests here]*<br><br>* typeset	
**Ethics Statement**<br><br>Enter an ethics statement for this submission. This statement is required if the study involved:<br><br>• Human participants<br>• Human specimens or tissue<br>• Vertebrate animals or cephalopods<br>• Vertebrate embryos or tissues<br>• Field research<br><br>Write "N/A" if the submission does not require an ethics statement.<br><br>General guidance is provided below. Consult the submission guidelines for detailed instructions. **Make sure that all information entered here is included in the Methods section of the manuscript.**	Not applicable

Powered by Editorial Manager® and ProduXion Manager® from Aries Systems Corporation

**Format for specific study types**<br><br>**Human Subject Research (involving human participants and/or tissue)**<br>• Give the name of the institutional review board or ethics committee that approved the study<br>• Include the approval number and/or a statement indicating approval of this research<br>• Indicate the form of consent obtained (written/oral) or the reason that consent was not obtained (e.g. the data were analyzed anonymously)<br><br>**Animal Research (involving vertebrate animals, embryos or tissues)**<br>• Provide the name of the Institutional Animal Care and Use Committee (IACUC) or other relevant ethics board that reviewed the study protocol, and indicate whether they approved this research or granted a formal waiver of ethical approval<br>• Include an approval number if one was obtained<br>• If the study involved *non-human primates*, add *additional details* about animal welfare and steps taken to ameliorate suffering<br>• If anesthesia, euthanasia, or any kind of animal sacrifice is part of the study, include briefly which substances and/or methods were applied<br><br>**Field Research**<br><br>Include the following details if this study involves the collection of plant, animal, or other materials from a natural setting:<br>• Field permit number<br>• Name of the institution or relevant body that granted permission	
**Data Availability**<br><br>Authors are required to make all data underlying the findings described fully available, without restriction, and from the time of publication. PLOS allows rare exceptions to address legal and ethical concerns. See the PLOS Data Policy and FAQ for detailed information.	Yes - all data are fully available without restriction

Powered by Editorial Manager® and ProduXion Manager® from Aries Systems Corporation

A Data Availability Statement describing where the data can be found is required at submission. Your answers to this question constitute the Data Availability Statement and **will be published in the article**, if accepted.<br><br>**Important:** Stating 'data available on request from the author' is not sufficient. If your data are only available upon request, select 'No' for the first question and explain your exceptional situation in the text box.<br><br>Do the authors confirm that all data underlying the findings described in their manuscript are fully available without restriction?	
**Describe where the data may be found in full sentences. If you are copying our sample text, replace any instances of XXX with the appropriate details.**<br><br>• If the data are **held or will be held in a public repository**, include URLs, accession numbers or DOIs. If this information will only be available after acceptance, indicate this by ticking the box below. For example: *All XXX files are available from the XXX database (accession number(s) XXX, XXX.).*<br>• If the data are all contained **within the manuscript and/or Supporting Information files**, enter the following: *All relevant data are within the manuscript and its Supporting Information files.*<br>• If neither of these applies but you are able to provide **details of access elsewhere**, with or without limitations, please do so. For example:<br><br>*Data cannot be shared publicly because of [XXX]. Data are available from the XXX Institutional Data Access / Ethics Committee (contact via XXX) for researchers who meet the criteria for access to confidential data.*<br><br>*The data underlying the results presented in the study are available from (include the name of the third party*	N/A

and contact information or URL).<br>• This text is appropriate if the data are owned by a third party and authors do not have permission to share the data.<br><br>* typeset	
Additional data availability information:	Tick here if your circumstances are not covered by the questions above and you need the journal's help to make your data available.

Manuscript Click here to access/download;Manuscript;Manuscript2.docx

Mapping of cost-effectiveness evaluations of the 9-Valent human papillomavirus (HPV) vaccine: Evidence from a systematic review

Rashidul Alam Mahumud[1*, 2, 3], Khorshed Alam[1,2], Syed Afroz Keramat[1,2,5], Gail Ormsby[6], Jeff Dunn[1,7,8], Jeff Gow[1,2,9]

Author Affiliations

[1]Health Economics and Policy Research, Centre for Health, Informatics and Economic Research, University of Southern Queensland, Toowoomba, Queensland-4350, Australia

[2]School of Commerce, University of Southern Queensland, Toowoomba, QLD-4350 Australia

[3]Health Economics and Financing Research, Health Systems and Population Studies Division, International Centre for Diarrhoeal Disease Research, Bangladesh (icddr,b), Dhaka-1212, Bangladesh.

[4]Health and Epidemiology Research, Department of Statistics, University of Rajshahi-6205, Bangladesh

[5]Economics Discipline, School of Social Science, University of Khulna, Khulna-9208, Bangladesh.

[6]Schoold of Health and Wellbeing, University of Southern Queensland, Toowoomba, Queensland-4350, Australia.

[7]Cancer Research Centre, Cancer Council Queensland, Fortitude Valley 4006, QLD, Australia.

[8]Prostate Cancer Foundation of Australia, St Leonards NSW 2065, Australia

[9]School of Accounting, Economics and Finance, University of KwaZulu-Natal, Durban, 4000, South Africa

*Corresponding author:

Health Economics and Policy Research, Centre for Health, Informatics and Economic Research, Faculty of Business, Education, Law and Arts, University of Southern Queensland, Toowoomba, Queensland-4350, Australia. ORCID: 0000-0001-9788-1868
E-mail: rashed.mahumud@usq.edu.au; rashidul.icddrb@gmail.com; Phone: +61746875657

Acknowledgements

The study is part of the first author's PhD research works. The PhD program was funded by the University of Southern Queensland, Australia. We would like to gratefully acknowledge the study participants and reviewers and editors of our manuscript.

Abstract

Introduction

The World Health Organization (WHO) recommends that programs for human papillomavirus (HPV) vaccination are established to be cost-effective before implementation. HPV vaccination is WHO recommended for girls aged 9–13 years old due to the high burden of cervical cancer. This review examined evidence of the cost-effectiveness evaluation of the 9-valent HPV vaccine within a global context.

Methods

Searches were performed until 31 July 2019 using two databases: PubMed and Scopus. A combined checklist (i.e., WHO, Drummond and CHEERS) was used to examine the quality of eligible studies. A total of 12 studies were eligible for review and nearly all were conducted in developed countries.

Results

Despite some heterogeneity in approaches to measuring cost-effectiveness, ten studies concluded that 9vHPV vaccination was cost-effective while two studies were not. The addition of adolescent boys into immunisation program was cost effective when vaccine price and coverage was comparatively low. When vaccination coverage for female was more than 75%, gender neutral HPV vaccination was less cost-effective than when targeting only girls aged 9–18 years. Multi cohort immunization approach was cost-effective in the age range of 9–14 years but the upper age limit at which vaccination was no longer cost-effective requires to be further evaluated. Most dominating parameters determined were duration of vaccine protection, time horizon, vaccine price, coverage, healthcare costs, efficacy and discounting rates.

Conclusions

These findings are anticipated to support policy-makers in extending HPV immunization programs on either switching to the 9-valent vaccine or inclusion of adolescent boys' vaccination or extending the age of vaccination. Further, this review also supports extending vaccination to low-resource settings where vaccine prices are competitive, donor funding is offered, cervical cancer burden is high and screening options are limited.

Introduction

Cervical cancer (CC) is both a leading cancer and the leading cause of cancer deaths in women globally [1]. Approximately 570,000 new cases of CC were diagnosed in 2018, composing 6.6% of all cancers in women [1]. The burden of CC is an alarming issue worldwide, especially in low- and middle-income countries (LMICs). Approximately 85% of CC cases and 90% of deaths from CC occur in LMICs [1]. Persistent infections with human papillomavirus (HPV) are a key cause of CC and is an established carcinogen of CC [2]. HPV is predominantly transmitted to reproductive-aged women through sexual contact [3]. Most HPV infections are transient and can be cleared up within a short duration, usually a few months after their acquisition. However, untreated HPV infections can continue and evolve into cancer in some cases. There are more than 100 types of HPV infections, and high-risk types develop into CC [4]. Thirteen high-risk HPV genotypes are known to be predominantly responsible for malignant and premalignant lesions of the anogenital area [5], and these are the leading causes of most aggressive CC [6]. Further, HPV is also responsible for the majority of anogenital cervical cancers, including anal cancers (88%), vulvar cancers (43%), invasive vaginal carcinomas (70%), and all penile cancers (50%) globally [4].

The burden of CC (i.e., high incidence and mortality rates) globally is preventable through the implementation of a primary prevention strategy such as vaccination [1]. There are vaccines that can protect common cancer-causing types of HPV and reduce the risk of CC significantly. Three types of HPV vaccines, namely bivalent (Cervarix), quadrivalent (Gardasil) and 9-valent vaccine (Gardasil-9), are currently available in the market. Unfortunately, as of March 2017, only 71 countries (37% of all countries) have introduced HPV vaccines in their national immunization programs for girls, and 11 countries (6%) for both sexes [2]. The first global recommendation on HPV vaccination was proposed by the World Health Organization's Strategic Advisory Group of Experts on Immunization in October 2008 [7], whereby HPV

vaccination was recommended for girls aged 9–13 years old. This recommendation was updated in April 2014 [8], with the emphasis to include extended 2-dose HPV immunization for girls aged 9–14 years, who were not immunocompromised. With the recent licensing of the 9-valent vaccine and the introduction of various HPV vaccination strategies, an update on the current recommendations of HPV vaccination are inevitable. The goals of the immunisation program are to reduce the acquisition and spread of HPV infections and to achieve optimum coverage through effective delivery systems. According to the underlying distribution of HPV infection types of CC, the 9vHPV vaccine builds population-level strong immunity against HPV-6, 11, 16, 18, 31, 33, 45, 52, and 58 infections [5] that cumulatively contribute approximately 89% of all CCs globally [9]. Considering the primary prevention of HPV infection, the 9vHPV vaccine is expected to reduce by an additional 10% the lifetime risk of diagnosis with CC in immunised cohorts compared with the 4vHPV vaccine and reduce CC by an additional 52% in non-vaccinated cohorts [10].

This review aims to update current evidence on the economic viability of HPV vaccination. In addition, this study aims to examine the cost-effectiveness of the 9-valent vaccine when boys are included and when age cohorts are varied, all within a global context. This review may be used as comprehensive evidence of general trends on the ongoing cost-effectiveness evaluation of HPV vaccine.

Materials and methods

Study design

Published original academic literature that examined the cost-effectiveness of 9vHPV vaccination were included in this systematic review. A wide type of study perspectives including societal and health systems perspectives were included. A search strategy was adopted considering all countries regardless of perspective or vaccine delivery strategy. A combined WHO [11], Drummond [12] and CHEERS [13] checklist was used to evaluate the quality of included studies.

Search strategy and sources

The literature search was performed by searching Scopus and PubMed to identify relevant articles following the inclusion criteria. Search inclusion terms included ‘economic evaluation’, ‘cost-effectiveness’, ‘analysis’, ‘human papillomavirus’, ‘HPV’, ‘vaccine’, ‘vaccinated’, ‘vaccination’, ‘cervical cancer’, ‘non-valent’, ‘9 or nine-valent’.

Study selection

Three authors of the review team independently examined the titles and abstracts of the articles that met the selection criteria. The existing academic literature in the cost-effectiveness of 9-*valent* HPV vaccination was searched. Exclusion of articles was based on: ‘not cost-effectiveness analyses, ‘insufficient cost and cost-effectiveness related data’, or ‘not using nine-valent HPV vaccine’. Language restrictions were not applied.

Data checking

The study strategy followed a number of checks to ensure consistency of approach, including a discussion about discrepancies within the study team. For each outcome and model input parameters, the authors identified the proportion of missing observations and compare them with data in the original publication. In addition, a range of checks was carried out for all included studies to ensure that all values were reasonable. Datasets were combined to form a new master dataset where model input assumptions and outcome-related parameters used in the original studies were included. Further, three authors independently assessed the analytical quality of the preliminary selected studies using appropriate tools for examining risk of bias. Disagreements on inclusions were resolved by discussion with a third review author.

Data extraction (selection and coding)

The study selection process was conducted in line with the PRISMA guidelines [14]. Data extraction was performed to develop a comprehensive data matrix which summarises the study characteristics such as authors, settings, perspective, threshold, outcome-related parameters and other necessary information.

Strategy for data synthesis

Two authors (RAM and SAK) independently reviewed the titles and abstract. Data from all eligible studies were extracted by the same two authors using a standardized data collection form. A matrix was developed to summarise the characteristics and findings of the studies.

Studies were characterized by incorporating four themes: (i) study used 9-valent HPV vaccine to examine the cost-effectiveness, (ii) target population demographic characteristics (e.g., gender-neutral and multiple age cohort immunisation), (iii) study perspectives, model and economic level of each country, and (iv) model input and outcome-related parameters.

To compare findings across the selected studies, incremental cost-effectiveness ratios (ICERs) and standardized cost-effectiveness were outlined. In terms of standardized cost-effectiveness scenarios, these studies used the heuristic cost-effectiveness threshold guided by the WHO [15], wherein an intervention or program was evaluated to be cost-effective if the ICER/DALYs averted was less than three times a country's annual per capita Gross Domestic Product (GDP). Further, the WHO constructed three broad decision rules: (i) an intervention or program was recommended as very cost-effective if ICER/DALYs averted <1 time GDP threshold; (ii) cost-effective if ICER/DALYs averted ≥ 1 time GDP threshold and ≤ 3 times GDP threshold; and (iii) not cost-effective if ICER/DALYs averted >3 times GDP threshold [16]. Examining whether an ICER offered by any strategy signifies value for money requires comparison to a cost-effectiveness threshold (CET). The CET refers to the health effects foregone (i.e., opportunity costs) related to resources being devoted to an intervention and consequentially being unavailable for other health-care priorities. Policy makers should be willing to invest their limited resources in the strategy offering the greatest health gains. CETs for the country with the lowest income in the world, borderline low/low-middle income, borderline low-middle/upper-middle income, and borderline high-middle/high income were estimated to be 1% to 51% GDP per capita, 4% to 51%, 11% to 51%, and 32% to 59%, respectively [17].

The review showed evidence in terms of methodological and current practices of cost-effectiveness evaluation studies such as determination of study research questions; the study perspective adopted, the duration of vaccine protection, time horizon and discount rate; explanation of model performed for data analysis; model input assumptions behind the estimation of associated costs and outcome parameters; reporting of ICERs; most dominant parameters of sensitivity analysis; examination of study conclusions and recommendations as well as financial disclosure of the selected studies.

Study characteristics

Four hundred and eighty one articles were yielded through the primary search, of which 78 articles were discarded because of duplication. Fifty one articles were considered for full-text

review after screening by title and abstract. Of these, 12 articles were eligible for the final review (Table 1). Three hundred fifty-two articles were excluded from this study following the inclusion criteria. The reasons for exclusion were: conference abstract (n = 58), reviews or editorials or commentary (n = 160), not cost-effectiveness evaluations (n = 60), did not use 9-valent vaccine (4v-HPV, 2v-HPV; n = 72) and insufficient information (n = 2). Finally, 12 articles were included in this review. The study selection procedure using the PRISMA flow method is shown in Figure 1.

<Insert Figure 1 and Table 1 in here>

Settings and funding

Single country studies mostly focused on high-income settings [4,18–29] (Table 2). However, a single study was found that covered two low-income countries (e.g., Kenya and Uganda) [30]. Eight studies were funded by research organisations [4,18,23,25–28,30], while two studies did not state funding sources [20,24]. The Bill and Melinda Gates Foundation was the sole funder of one study [30] and three studies were funded by the Centre for Disease Control (CDC) [20,25,28]. Further, five studies were conducted in United States [20,21,24,26,28], one study was conducted in each of Germany [27], Italy [4], China [22], Australia [29], Austria [18] and Canada [23]. Low resource countries mostly depend on external funding agency for HPV vaccine programs, hence these countries may have less impetus for cost-effectiveness studies to inform local decision making as priorities are driven by external considerations.

<Insert Table 2 in here>

Study questions and comparator

Most studies (n = 8) investigated the cost-effectiveness of introducing HPV vaccination to preadolescent girls aged 12 or younger [4,18,19,22,23,26,28–30]. Four studies assessed vaccinating 12 years or older girls [20,21,24,27]. All studies investigated vaccination either as an addition to existing screening programs or (more commonly) as opportunistic preventive programs or none at all. Further, most studies considered a range of vaccination and screening options to find the most cost-effective combination.

Analytical model

Nine studies used a dynamic economic model for examining the cost-effectiveness of HPV vaccination programs [4,18–22,24–29], two studies used a static model [23,30], and one study

used a Markov model for analytical exploration [22] (Table 2). However, some studies did not explicitly account for the pathologic transition from HPV acquisition to HPV-associated disease [4,20–22,29], pathologic transition [4,27] and herd immunity [18,20,21,23,24,28].

Thresholds and perspectives

In terms of the cost-effectiveness scenario, four studies used the heuristic cost-effectiveness threshold proposed by the WHO. These studies used either one or three times GDP per capita [22,23,28,30]. The majority of studies adopted their own local thresholds (e.g., willingness to pay) while three studies considered both thresholds of GDP per capita and willingness to pay [22,28,30]. Apart from these studies, seven studies undertook an evaluation from a societal perspective [20,21,23,26–28,30], and four studies utilised a health system perspective [4,22,24,29]. Several studies used a societal perspective and included all vaccination costs, relevant direct medical costs, and gains in quality and length of life without regard to who incurred the costs or who received the benefits (Table 2). However, these selected studies reported little about the indirect costs and productivity losses which are significant from a societal perspective.

Vaccine coverage

The assumptions on vaccine coverage are significant in influencing the potential impact of HPV vaccine on HPV related diseases. Four selected studies assumed a vaccination coverage rate of 90% or above [22,26,27,30]. The vaccine coverage might be varied in terms of study settings as well as from a gender point of view. Among the selected studies, three studies considered a vaccine coverage rates of 26-60% for females and 25-40% for males [18,20,21], and other three studies considered a 46-80% vaccine coverage rate [23,24,29]. Three studies grouped coverages based on gender, with vaccination coverage assumed for females (25-60%) and for males (11-40%) [20,21,28]. The remaining study did not specify the vaccine coverage rate to be used [28].

Vaccine efficacy

Most studies considered vaccine efficacy rate ranged from 95-100% against HPV infections except the study of Simms et al. (2016) [29], which considered a vaccine efficacy rate of only 59%. The study conducted in two East African Countries (Kenya and Uganda) used a 100% vaccine efficacy rate in case of 9vHPV [30]. Most studies (n=10) used a 95% vaccine efficacy rate [4,18–28].

Number of vaccine dose and delivery route

Eight studies used a three-dose schedule of nine-valent vaccine. Most studies (6/8) were conducted in developed countries [20–25,28,30] and the other two studies were conducted in low- and middle-income countries [22,30]. Further, one study conducted in the United States [25] used both 2- and 3-dose vaccines. Diverse vaccine delivery routes were evidenced across the studies. Nine studies used the vaccine delivery route of a national immunisation program for the target population [4,20–25,29,30]. Two studies conducted in Austria [18] and United States [28], used a universal immunisation strategy to deliver the vaccine. Only one cost-effectiveness exploration of 9vHPV vaccine was conducted in Germany [27] and it used a vaccine delivery route through social health insurance.

Duration of vaccine protection, herd effect and discounting rate

Most studies (11/12) assumed lifelong vaccine protection while only one study assumed a shorter duration of protection of 20 years [23]. Half of the studies specified herd immunity due to vaccination [18,20,21,23,24,28]. The remaining six studies did not consider the indirect effect of vaccination. Regarding the discount rate, majority of the studies (11/12) used 3% discount rate, while one study considered a 5% discount rate to adjust for future values in terms of economic value and health [29].

Risk of bias and quality of included studies

Risk of bias was examined using the Consensus Health Economic Criteria (CHEC) list, a checklist that can be used to critically evaluate published economic evaluations [31]. Table 3 showed the extent to which the reviewed studies conformed with the standards for reporting economic evaluations based on the WHO guidance [11], Drummond [12] and CHEERS [13]. All studies clearly identified the study question, intervention(s), and comparator(s). A relatively high proportion of studies reported their study perspectives (12/12; 100%), time horizon (12/12; 100%) and discounting rates (12/12; 100%). Most studies performed sensitivity analyses (11/12; 92%) to assess the robustness of their findings. Almost all studies also clearly described the measurements and the assumption behind the calculation costs (11/12, 92%). The choice of model used was justified in majority studies (12/12; 100%), as a high proportion of these studies adopted dynamic transmission model to capture herds immunity. The currency and price data were reported in most studies too (12/12; 100%). Ten (83%) out 12 studies disclosed their funding sources. However, only 8 studies (67%) reported the measurement of

effectiveness from synthesis-based estimates, either through the combination of several randomized trials or the use of systematic reviews.

<Insert Table 3 in here>

Results

Ten studies concluded that their evaluation of 9vHPV vaccination was found to be cost-effective (Table 4) while the remaining two studies did not find cost-effectiveness [20,21]. Further, five studies exhibited a ‘very cost-effective’ decision [4,22,23,27,30] and four studies found ‘cost-savings’ [18,20,26,28]. In the context of high-income countries (e.g., Canada and Austria), introduction of 9vHPV vaccination was a cost-effective decision to prevent cervical cancer of adolescent girls, as the incremental cost of vaccine compared with 9vHPV vaccine was less than US$23-US$47. However, in low and middle-income countries (e.g., Kenya and Uganda), the ICER of 9-valent vaccine must not be priced over US$8.40-US$9.80 [23,30]. Two USA studies concluded that the cost-effectiveness exploration of 9-valent vaccine was more likely to be ‘cost-saving’ regardless of cross-protection assumption [20,28]. Most studies used ‘quality-adjusted life year’ (QALYs) as the measurement unit of cost-effectiveness. In addition, selected studies explored the cost-effectiveness decision using WTP thresholds that depended on country settings. When country specific vaccine prices are used then the cost-effectiveness decision changes. For example, two studies conducted in the USA, considered two different vaccine prices per dose, US$162.74 and US$174, respectively. However, both studies confirmed that the introduction of 9-valent vaccine was not cost-effective using their model input assumptions. Four studies reported cost-effectiveness of 9-valent vaccine for gender-neutral approaches [18,20,24,28] and three studies found it a ‘cost-effective’ or ‘cost-saving’ decision [18,20,28]. The remaining eight studies suggested vaccinating girls only. In terms of key drivers of cost-effectiveness, several dominating parameters were identified in this review such as duration of vaccine protection [18,23,29], time horizon [21], vaccine price [4,20,21,23,24,27–29], healthcare costs [26], vaccine efficacy [23,26], vaccine coverage [23,26] and discounting rates [18,23,27,30].

<Insert Table 4 in here>

Discussion

The HPV vaccination is one of the cornerstones of CC prevention worldwide. This study explored the cost-effectiveness of 9-valent HPV vaccination, drawing on 12 cost-effectiveness

evaluations in order to inform and expand knowledge of the potential influence of the next generation of HPV vaccines. Most studies were conducted in developed countries while one study was performed in an LMIC. However, in the context of LMICs, the incidence of cervical cancer is an alarming public health concern, which warrants an increase in studies which can be extremely useful to influence local decision making [32]. The economic viability of gender-neutral 9-valent HPV vaccination was confirmed by three of the selected studies [18,20,28]. Cost-effectiveness exploration depends on the coverage of vaccination from the perspective of gender. For example, if the vaccine coverage for female recipients is 80% or above, the majority of the anogenital CC include vulvar cancers, invasive vaginal carcinomas cancers in female could be prevented. As a result, introduction of 9-valent vaccination for boys is relatively less important compared with girls due to the high economic costs involved without the additional benefits gained as per the female population reduction in CC, both from the societal and health system perspectives. Therefore, achieving optimal coverage of vaccination in females should remain a priority. This is of primary significance for LMICs settings since it is more effective and economically viable to prevent CC in females. However, it is also important to note that past studies paid little attention to the broader benefits of vaccination among male cohorts to prevent penile, anal, and oropharyngeal cancers. Exclusion of these diseases related to males may undermine the effectiveness of reducing CC. Gender-neutral vaccination might have several benefits including herd protection for boys. Moreover, it may provide indirect protection to unvaccinated women and direct protection to homosexual men. Therefore, this vaccination strategy should be further considered in country-level immunization programs by underlining other parameters including disease burden, sexual behaviour in a country (e.g., homosexual intercourse), equity, budget impact, and affordability.

Despite different methodologies and various assumptions, most studies were consistent in their conclusion that multiple age cohort vaccination was economically viable. Nevertheless, there was an upper age limit at which HPV vaccination was no longer cost-effective, and should be interpreted cautiously as several studies evaluated the cost-effectiveness in a single age range only and did not compare to the next age range in a progressive manner. Subsequently, this could result in an overestimation of the cut-off age range for vaccination. The protection duration from vaccination has a large impact on the cost-effectiveness of multi-cohort vaccination, with most studies assuming life-long protection. Therefore, the use of ICERs based on the conventional evaluation of 10-year protection may be more representative of real-life effectiveness rather than the use of ICER based on lifetime protection. The cost-

effectiveness of HPV vaccination is also dependent upon the levels of vaccine coverage, compliance, and vaccine price.

Most models presumed a high coverage of vaccination, e.g., assuming that 70% of the target population will receive full doses of vaccination. However, not everyone who initiates the vaccination completes full doses (i.e., two or three doses) within the recommended time frame. Therefore, cost-effectiveness evaluation may underestimate or overestimate the actual costs. The analytical model outcomes in terms of herd immunity is only hypothetical unless the coverage level increases among the study cohort. Further, it is also indeterminate how non-compliance may consequently influence vaccine efficacy, effectiveness and duration of protection [55]. Model input assumptions regarding the 9-valent vaccine price also influence the cost-effectiveness outcomes observed. Prices for 9-valent vaccine are currently not specified, particularly, in lower-income countries. Hence, the cost-effectiveness of 9-valent vaccine is still indeterminate and there is no exclusive evidence of greater cost-effectiveness than the older licensed HPV vaccines. In fact, HPV vaccination is only cost-effective under the assumption of the lowest price of 9-valent vaccine.

Therefore, once the 9-valent vaccine price is fixed, including support by the GAVI vaccine-alliance, reassessment of cost-effectiveness of 9-valent vaccine is necessary. Another model input assumption that may influence the cost-effectiveness is the inclusion or exclusion of herd immunity effects based on the type of model acceptance. Two studies [23,30] constituted the static model as an analytical exploration which did not confirm herd immunity effects. Generally, the cost-effectiveness evaluations of HPV vaccine should use a dynamic model for exploration because economic evaluations for primary prevention strategy should be determined by societal benefits (e.g., indirect impacts on population were not immunised) rather than individual demands [33]. However, the application of a static model in these two studies may underestimate or overestimate the benefits of vaccination. If an HPV vaccination program is exhibited to be cost-effective considering a static model for analytical exploration, it is anticipated to be even very cost-effective when a dynamic model is considered [33].

A cost-effectiveness threshold is commonly fixed so that the interventions or programs that appear to be a comparatively good or very good value for money can be determined. There are several types of threshold. The majority of the studies used the cost-effectiveness demand side-threshold (e.g. willingness-to-pay). In health-related explorations, a willingness-to-pay threshold signifies an evaluation of what a consumer of health care might be prepared to pay

for the health benefit – given other competing demands on that consumer's resources. There are also supply-side thresholds that resource allocation mechanism takes into account. For example, estimates of health status are predetermined since when an insurance company or other provider spends some of its available budget on a new intervention it is therefore required to decrease its funding of previous interventions. In considering the choice of the type of cost-effectiveness threshold to use, the concept of opportunity cost may be the one most relevant to providers who are primarily concerned with using available resources to maximise improvements in health status. In considering the implementation of a new intervention, decision-makers need estimates of both the health that might be gained elsewhere through the alternative use of the resources needed for the new intervention and the health that is likely to be lost if the new intervention is not used.

This review has some limitations. The cost-effectiveness evaluation based on GDP based thresholds of 1–3 times of GDP per capita lacks country specificity and has little meaning for country-level decision making [34]. It is uncertain whether this threshold truly reflects the country's affordability or societal willingness to pay for additional health gains. Additionally, GDP is originally intended to measure the experience of people residing in urban areas and thus, it may not actually reflect the experience of the entire population in a country, especially those living in rural areas. Apart from an economic standpoint, other factors should be considered for the national immunization program, such as budget availability, political issues, cultural influences and availability of healthcare workforce.

Conclusions

Current evidence does not show conclusive proof of greater cost-effectiveness of the new 9-valent vaccine. The inclusion of adolescent males in HPV vaccination programs is cost-effective if vaccine price or coverage of females is low and if the HPV-associated male diseases are also considered. Multiple age cohort vaccination strategy is likely to be cost-effective in the age range of 9–14 years, but the upper age limit at which HPV vaccination is no longer cost-effective needs to be further evaluated. Vaccine coverage, price, duration of protection and discount rates are important parameters for consideration in the uptake of HPV vaccination. Nonetheless, present study findings may be used as an evidence to policy-makers and healthcare providers in making recommendations for HPV national immunization programs on the new 9-valent vaccine or inclusion of adolescent boys' vaccination or extending the age of

immunization, but it should not divert resources from vaccinating the primary target population of girls aged 12 years or from effective cervical cancer screening programs.

References

1. World Health Organization (WHO). National cancer contro programmes: Cervical cancer statistics. 2019 [cited 29 Aug 2019]. Available: https://www.who.int/cancer/prevention/diagnosis-screening/cervical-cancer/en/

2. Forman D, Lortet-Tieulent J, de Martel C, Ferlay J, Franceschi S, Plummer M, et al. Global burden of human papillomavirus and related diseases. Vaccine. 2012;30: F12–F23. doi:10.1016/j.vaccine.2012.07.055

3. Centers for Disease Control (CDC). Genital HPV infection—fact sheet. Centers for disease control and prevention. 2015 [cited 14 Feb 2019]. Available: http://www.cdc.gov/std/hpv/stdfact-hpv.htm

4. Mennini FS, Bonanni P, Bianic F, Waure C, Baio G, Plazzotta G, et al. Cost-effectiveness analysis of the nine-valent HPV vaccine in Italy. Cost Eff Resour Alloc. 2017;15: 1–14. doi:10.1186/s12962-017-0073-8

5. Guan P, Howell-Jones R, Li N, Bruni L, De Sanjosé S, Franceschi S, et al. Human papillomavirus types in 115,789 HPV-positive women: A meta-analysis from cervical infection to cancer. Int J Cancer. 2012;131: 2349–2359. doi:10.1002/ijc.27485

6. Li N, Franceschi S, Howell-Jones R, Snijders PJF, Clifford GM. Human papillomavirus type distribution in 30,848 invasive cervical cancers worldwide: Variation by geographical region, histological type and year of publication. Int J Cancer. 2011;128: 927–935. doi:10.1002/ijc.25396

7. World Health Organization (WHO). Human papillomavirus vaccines: WHO position paper. Wkly Epidemiol Rec. 2009;84: 118–131.

8. World Health Organization (WHO). Human papillomavirus vaccines: WHO position paper, October 2014. Wkly Epidemiol Rec. 2014;89: 465–491.

9. Serrano B, Alemany L, Tous S, Bruni L, Clifford GM, Weiss T, et al. Potential impact of a nine-valent vaccine in human papillomavirus related cervical disease. Infect Agent Cancer. 2012;7: 1–13. doi:10.1186/1750-9378-7-38

10. Simms KT, Laprise JF, Smith MA, Lew J Bin, Caruana M, Brisson M, et al. Cost-effectiveness of the next generation nonavalent human papillomavirus vaccine in the context of primary human papillomavirus screening in Australia: A comparative modelling analysis. Lancet Public Heal. 2016;1: e66–e75. doi:10.1016/S2468-2667(16)30019-6

11. Walker DG, Hutubessy R, Beutels P. WHO Guide for standardisation of economic evaluations of immunization programmes. Vaccine. 2010;28: 2356–2359. doi:10.1016/j.vaccine.2009.06.035

12. Drummond MF, Jefferson T. Guidelines for authors and peer reviewers of economic submissions to the BMJ. The BMJ economic evaluation working party. BMJ. 1996;313: 275–83.

13. Husereau D, Drummond M, Petrou S, Carswell C, Moher D, Greenberg D, et al. Consolidated health economic evaluation reporting standards (CHEERS)-explanation and elaboration: A report of the ISPOR health economic evaluation publication guidelines good reporting practices task force. Value Heal. 2013;16: 231–250. doi:10.1016/j.jval.2013.02.002

14. Moher Bd, Liberati A, Tetzlaff J, Altman DG. Preferred reporting items for systematic reviews and meta-analyses: The PRISMA statement. PLoS Med. 2009;6: e1000097. doi:10.1371/journal.pmed.1000097

15. WHO Commission on Macroeconomics and Health. Macroeconomics and health: Investing in health for economic development. Report of the Commission on Macroeconomics and Health. 20 Avenue Appia, 1211 Geneva 27, Switzerland; 2001. Available: http://whqlibdoc.who.int/publications/2001/924154550x.pdf

16. Edejer TT-T, Baltussen R, Adam T, Hutubessy R, Acharya A, Evans D., et al. Making choices in health: WHO guide to cost-effective analysis. 20 Avenue Appia, 1211 Geneva 27, Switzerland; 2003. Report No.: 9241546018.

17. Woods B, Revill P, Sculpher M, Claxton K. Country-level cost-effectiveness thresholds: Initial estimates and the need for further research. Value Heal. 2016;19: 929–935. doi:10.1016/j.jval.2016.02.017

18. Boiron L, Joura E, Largeron N, Prager B, Uhart M. Estimating the cost-effectiveness profile of a universal vaccination programme with a nine-valent HPV vaccine in Austria. BMC Infect Dis. 2016;16: 1–15. doi:10.1186/s12879-016-1483-5

19. Franco A, Barrull C, Perulero N, Torné A, San-Martin M, López N, et al. Epidemiologic and economic burden of HPV diseases in Spain: implication of additional 5 types from the 9-valent vaccine. Infect Agent Cancer. 2018;13: 1–10. doi:10.1186/s13027-018-0187-4

20. Chesson HW, Markowitz LE, Hariri S, Ekwueme DU, Saraiya M. The impact and cost-effectiveness of nonavalent HPV vaccination in the United States: Estimates from a simplified transmission model. Hum Vaccines Immunother. 2016;12: 1363–1372. doi:10.1080/21645515.2016.1140288

21. Chesson HW, Laprise JF, Brisson M, Markowitz LE. Impact and Cost-effectiveness of 3 Doses of 9-Valent Human Papillomavirus (HPV) vaccine among US females previously vaccinated with 4-valent hpv vaccine. J Infect Dis. 2016;213: 1694–1700. doi:10.1093/infdis/jiw046

22. Mo X, Gai Tobe R, Wang L, Liu X, Wu B, Luo H, et al. Cost-effectiveness analysis of different types of human papillomavirus vaccination combined with a cervical cancer screening program in mainland China. BMC Infect Dis. 2017;17: 1–12. doi:10.1186/s12879-017-2592-5

23. Drolet M, Laprise JF, Boily MC, Franco EL, Brisson M. Potential cost-effectiveness of the nonavalent human papillomavirus (HPV) vaccine. Int J Cancer. 2014;134: 2264–2268. doi:10.1002/ijc.28541

24. Chesson HW, Meites E, Ekwueme DU, Saraiya M, Markowitz LE. Cost-effectiveness of nonavalent HPV vaccination among males aged 22 through 26 years in the United States. Vaccine. 2018;36: 4362–4368. doi:10.1016/j.vaccine.2018.04.071

25. Laprise JF, Drolet M, Boily MC, Jit M, Sauvageau C, Franco EL, et al. Comparing the cost-effectiveness of two- and three-dose schedules of human papillomavirus vaccination: A transmission-dynamic modelling study. Vaccine. 2014;32: 5845–5853. doi:10.1016/j.vaccine.2014.07.099

26. Laprise JF, Markowitz LE, Chesson HW, Drolet M, Brisson M. Comparison of 2-dose and 3-dose 9-valent human papillomavirus vaccine schedules in the United States: A cost-effectiveness analysis. J Infect Dis. 2016;214: 685–688. doi:10.1093/infdis/jiw227

27. Largeron N, Petry KU, Jacob J, Bianic F, Anger D, Uhart M. An estimate of the public health impact and cost-effectiveness of universal vaccination with a 9-valent HPV vaccine in Germany. Expert Rev Pharmacoeconomics Outcomes Res. 2017;17: 85–98. doi:10.1080/14737167.2016.1208087

28. Brisson M, Laprise JF, Chesson HW, Drolet M, Malagón T, Boily MC, et al. Health and economic impact of switching from a 4-Valent to a 9-valent HPV vaccination program in the United States. J Natl Cancer Inst. 2016;108: 1–9. doi:10.1093/jnci/djv282

29. Simms KT, Laprise JF, Smith MA, Lew J Bin, Caruana M, Brisson M, et al. Cost-effectiveness of the next generation nonavalent human papillomavirus vaccine in the context of primary human papillomavirus screening in Australia: a comparative modelling analysis. Lancet Public Heal. 2016;1: e66–e75. doi:10.1016/S2468-2667(16)30019-6

30. Kiatpongsan S, Kim JJ. Costs and cost-effectiveness of 9-valent human papillomavirus (HPV) vaccination in two east african countries. PLoS One. 2014;9: 1–6. doi:10.1371/journal.pone.0106836

31. Evers S, Goossens M, de Vet H, van Tulder M, Ament A. Criteria list for assessment of methodological quality of economic evaluations: Consensus on Health Economic Criteria. Int J Technol Assess Health Care. 2005;21: 240–245. doi:10.1017/s0266462305050324

32. Thiboonboon K, Santatiwongchai B, Chantarastapornchit V, Rattanavipapong W, Teerawattananon Y. A systematic review of economic evaluation methodologies between resource-limited and resource-rich countries: A case of rotavirus vaccines. Appl Health Econ Health Policy. 2016;14: 659–672.

33. Ng SS, Hutubessy R, Chaiyakunapruk N. Systematic review of cost-effectiveness studies of human papillomavirus (HPV) vaccination: 9-Valent vaccine, gender-neutral and multiple age cohort vaccination. Vaccine. 2018;36: 2529–2544. doi:10.1016/j.vaccine.2018.03.024

34. Bertram MY, Lauer JA, De Joncheere K, Edejer T, Hutubessy R, Kieny MP, et al. Cost-effectiveness thresholds: Pros and cons. Bull World Health Organ. 2016;94: 925–930. doi:10.2471/BLT.15.164418

Legend Figure

Figure 1. PRISMA flow-chart for systematic review of studies

Figure 1

Click here to access/download;Figure;Figure 1.tif

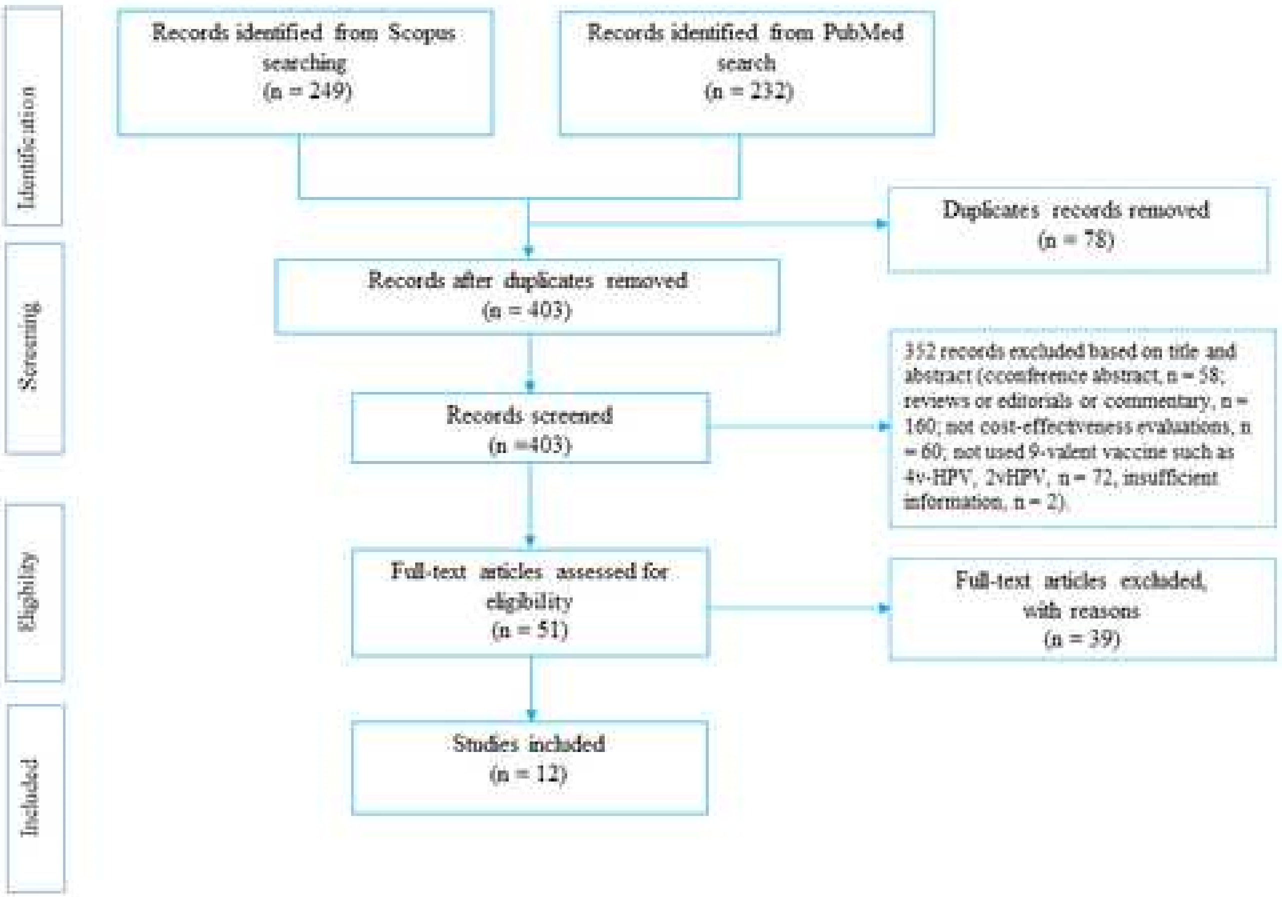

Table 1. Characteristics of twelve included cost-effectiveness of 9-valent vaccine

Characteristics	Number of studies (n)	Percentage (%)
Selected articles	12	100
Year of publication		
2014	2	17
2016	7	58
2017	2	17
2018	1	8
Name of Journal		
BMC Infectious Diseases	2	17
Cost Effectiveness and Resource Allocation	1	8
Expert Review of Pharmacoeconomics & Outcomes Research	1	8
Human Vaccines & Immunotherapeutics	1	8
International Journal of Cancer	1	8
Lancet Public Health	1	8
PLOS ONE	1	8
The Journal of Infectious Diseases	2	17
Vaccine	1	8
Journal of the National Cancer Institute	1	8
Study setting		
Australia	1	8
Austria	1	8
Canada	1	8
China	1	8
Germany	1	8
Italy	1	8
Kenya and Uganda	1	8
United States	5	42
Main location of first author		
Research institute	8	67
Research group	1	8
Hospital or University	3	25
Conflict of interest		
Yes	6	50
No	6	50

Table 2. Characteristics of the selected studies

Author	Study settings	Economic category	Target age cohort	Sex of cohort	Vaccine delivery route	No of doses	Type of model	Threshold	Perspective	Time horizon (year)	Discount rate	Sensitivity analysis	Most sensitive parameter
Kiatpongsan et al. [29]	Kenya and Uganda	LMIC	9 years	Female	NIP	3	Static	GDP and WTP	Societal	ns	3%	One-way	Discount rate
Laprise et al. [25]	United States	HI	9–14 years	Female	NIP	2 & 3	Dynamic	WTP	Societal	100	3%	One-way	Vaccine efficacy, screening method, and healthcare costs, vaccine coverage
Largeron et al. [26]	Germany	HI	12-17 years	Female	SHI plans	2	Dynamic	WTP	Societal	100	3%	One-way	Discounted rate, vaccine price
Mennini et al. [4]	Italy	HI	12 years	Female	NIP	2	Dynamic	WTP	Health system	100	3%	One-way	Vaccine price
Mo et al. [21]	China	MI	12 years	Female	NIP	3	Markov	GDP and WTP	Health system		3%	One-way	
Simms et al. [28]	Australia	HI	12 years	Female	NIP	2	Dynamic	WTP	Health system	20	5%	One-way	Vaccine price and vaccine duration of protection
Boiron et al. [17]	Austria	HI	9-year	Gender-neutral	Universal	2	Dynamic	WTP & GDP	Health system	100	3%	one-way	Discount rates and duration of protection
Brisson et al. [27]	United States	HI	9-year	Gender-neutral	Universal	3	Dynamic	WTP	Societal	70	3%	One-way	Vaccine price
Chesson et al. [19]	United States	HI	Female: 12 to 26 years, and male:12 to 21 y	Gender-neutral	NIP	3	Dynamic	WTP	Societal	100	3%	one-way	Vaccine price, Time horizon
Chesson et al. [20]	United States	HI	Female:13-18years	female	NIP	3	Dynamic	WTP	Societal	100	3%	One-way, Multi-way	Vaccine price
Chesson et al. [23]	United States	HI	Female: 12 to 26 years, and male:12 to 21 y	Gender-neutral	NIP	3	Dynamic	WTP	Health system	100	3%	One-way, Multi-way	Vaccine price
Drolet et al. [22]	Canada	HI	10 years	Female	NIP	3	Static	GDP	Societal	70	3%	One-way, Multi-way	Duration of protection, vaccine efficacy, vaccine price, discount rate

Note: Statutory health insurance (SHI) plans, NIP = National Immunisation Program, WTP = Willingness to pay,

Table 3. Extent to which included studies met standard reporting recommendations

Explained recommendations	Number of studies fulfilling	Percentage (%)
Research question or objective clearly stated	10/12	83
Described intervention and comparator	10/12	83
Exploration of effectiveness reported	11/12	92
Single study-based estimates	8/12	67
Synthesis-based estimates	10/12	83
Assumption of costs and outcomes specified	11/12	92
Currency and price data reported	12	100
Choice of model justified	12	100
Perspective specified	12	100
Time horizon specified	12	100
Discounting rates specified	12	100
Calculated and reported ICER or cost-saving	12	100
Sensitivity analysis performed	11/12	92
Conclusions follow from the data reported	12	100
Disclosed funding source(s)	10/12	83

Table 4. Summary of the results of the selected studies

Author	Vaccine efficacy	Vaccine coverage	Duration of vaccine protection	Herd effect	Vaccine price per dose	Unit of cost-effectiveness	GDP per capita	Incremental cost-effectiveness ration (ICER)	Conclusion or recommendation	Study funder
Kiatpongsan et al. [29]	100%	100%	Lifetime	No	US$ 90.25	QALYs	Kenya = $1,349.97, Uganda = $ 674.05	Very cost-effective if additional cost of 9vHPV vaccine per course ≤ $9.8 in Kenya & ≤ 8.4 in Uganda	Very cost-effective for both countries (Kenya & Uganda)	The Bill and Melinda Gates Foundation
Laprise et al. [25]	95%	90%	Lifetime	No	US$ 158	QALYs		Cost saving to US$ 500	Cost saving	CDC
Largeron et al. [26]	96%	90%	Lifetime	No	€ 140	QALYs	£30,000	€ 329 / QALY	Highly cost-effective	Sanofi Pasteur MSD (SPMSD).
Mennini et al. [4]	96%	90%	lifelong	No	€ 80.00	QALYs	€ 40,000	€ 10,463 / QALY	Highly cost-effective	Sanofi Pasteur MSD
Mo et al. [21]	96.7%	20%	lifetime	No	USD 149.03	QALYs	USD 23,880	US$ 5,768 / QALY	- Highly cost-effective with screening 1 + 9vHPV,<br>- Cost-effective with screening 2 + 9vHPV	The Japan Society for the Promotion of Sciences, the National Centre for Child Health and Development, and the Chinese Natural Sciences Foundation
Simms et al. [28]	59%	70%	lifelong	No	ns	QALYs	AUD 30,000	Cost-eff ectiveness if the additional cost per dose is US$18–28	Cost-effectiveness	National Health and Medical Research Council, Australia
Boiron et al. [17]	98%	Female: 60%, Male: 40%	Lifelong	Yes	US$ 147.15	QALYs	US$ 44,767.35	Cost-saving at vaccine prince up to US$ 166.77	Cost-saving	Sanofi Pasteur MSD
Brisson et al. [27]	95.0%	Not stated	Lifelong	Yes	US$ 158	QALYs	US$ 48,373.88	Cost-saving regardless of cross-protection assumptions	Cost-saving if additional cost of vaccine per dose < US$ 13	CDC, Canadian Research Chair Program
Chesson et al. [19]	95.0%	Female: 25.8% Male: 11.7%	Lifelong	Yes	US$ 162.74	QALYs	US$ 52,787.03	Cost-saving regardless of cross-protection assumptions (<$0)	Cost-saving	Not stated
Chesson et al. [20]	95.0%	Female: 46% Male : 25%	Lifelong	Yes	US$ 162.74	QALYs	US$ 52,787.03	US$ 111,446 / QALY	Not cost-effective	CDC, Canada Research Chair Program, Canadian Institute for Health Research
Chesson et al. [23]	95.0%	46%	Lifelong	Yes	US$ 174	QALYs	US$ 52,787.03	US$ 228,800 / QALY	Not cost-effective	Not stated
Drolet et al. [22]	95.0%	80%	20years	Yes	US$ 90.25	QALYs	US$ 50,440.44	US$ 11,593 /QALY	Very cost-effective if additional cost of vaccine per dose ≤ US$ 22.80	Canadian Research Chair Program

PRISMA Checklist

Click here to access/download
Supporting Information
PRISMA 2009 checklist_.doc
